# Supplementary material for: Factors associated with Macrosomia in public hospitals of Mekelle City, Northern Ethiopia: A multi-center study
Source: PLoS One. 2025 Jun 3;20(6):e0325541. doi: 10.1371/journal.pone.0325541 (PMC12132980; doi:10.1371/journal.pone.0325541)
Supplement: Attachment — Submitted filename: Review report.pdf [file pone.0325541.s002.pdf]

Review report

It is a great pleasure for me to review the manuscript of this demanding public health problem conducted in the Tigray region of northern Ethiopia. However, this manuscript is not suitable for publication in its current standing, as it needs major revision in the following sections and I hope the authors will be benefitted from this feedback to further improve the manuscript for scientific publication.

Title

The title requires revisions, as the authors have indicated it follows a case-control design. Therefore, the phrase **"factors associated"** is not the most suitable epidemiological terminology for this type of study design. Furthermore, the title should be revised to accurately reflect the study design and ensure appropriate epidemiological terms.

Abstract and introduction section.

The background/introduction section requires enhancement as it currently lacks depth and does not clearly articulate the problem at hand. It is important to establish a strong rationale for conducting this research. **What unique contributions does this study bring compared to previous work, and what justifications can be provided?** Although the background states that there is limited knowledge about the factors leading to macrosomia in Northern Ethiopia, it is essential to acknowledge that existing studies have already documented these factors. Recognizing this literature will add valuable context to the research.

e.g.,

1. https://bmcpediatr.biomedcentral.com/articles/10.1186/s12887-017-0901-1.
2. https://pubmed.ncbi.nlm.nih.gov/31262264/.
3. https://journals.sagepub.com/doi/10.1177/03000605221132028?icid=int.sj-full-text.similar-articles.6.

Objectives: This section needs revision based on the comments given above.

Results: This section needs significant revision, please write the adjusted measures of effect (AOR with its 95% CI). Likewise, try to make the interpretation as meaningful as possible. How

do you **interpret risk and preventive odds ratio**? As the age of the mother increases by one year, the odds of macrosomia were found to increase by 12%?? The odds of macrosomia increased by 36% and 26%?? Please revise the interpretation by considering these recommendations.

Study design, area, and Period

- The authors have focused exclusively on Mekelle City for their research, raising questions about the decision not to include other general hospitals in the region, such as those in Axum, Adigrat, Maichew, Abiyi Adi, Suhul Shire, and Kidiste Mariam, among others. This choice prompts a discussion about the factors influencing the selection of Mekelle city over the six or seven zones that make up the region. Understanding the rationale behind this focus could provide insight into the research objectives and outcomes.
- The previously conducted studies in the Tigray region were also in Mekelle, why this study is also in Mekelle without clear added value?
- Mekelle is 783 kilometers from Addis Ababa
- Put reference for the mentioned statements

Eligibility criteria

- The authors mentioned only the exclusion criteria, what about the inclusion criteria?
- Macrosomia- is the weight of the newborn greater than or equal 4000g [1,2,27]. Use only a single reference.
- **predictors of macrosomia vs determinants vs factors associated, which one are the authors studying?**

Data collection tools and procedures

- Put reference for the data collection tool, from which literatures did the authors use to develop the tool?
- The collected data were coded, entered, cleaned, and analyzed using SPSS version 23. Why the authors fail to use data entry software so that they can minimize data entry errors. Any explanation?
- In the data analysis plan, please mention that the crude and adjusted measures of effect (COR and AOR), how do the authors select candidate variables from bivariate to

multivariable model? Did the authors include those variables which were statistically non-significant but clinically important in their model?

- Is it possible to calculate percent if the denominator is less than 100, e.g., the total number of cases is 92, which is <100. Is it appropriate say “Male newborns accounted for 78.3% of cases”?? Please mention only the numbers if the denominator is less than 100. Do not calculate percentages.
- What is the interpretation for the significant difference in the baseline sociodemographic characteristics of the cases and controls? Similarly, the cases and controls were not comparable based on their Obstetric, anthropometrical, and health-related characteristics? If they were not comparable from the beginning, do you think the findings are reliable and generalizable?
- **Predictors of Macrosomia: This is not appropriate ate epidemiologic term for your study design. Please revise and present appropriately.**
- No need to present the multicollinearity, the omnibus test of model coefficients was significant, Cox and Snell residuals, Nagelkerke’s R2 and the ROC curve analysis in the result section.
- However, the Hosmer and Lemeshow test results should be presented in the final regression table.
- The final regression table should be presented again containing the non-significant variables too. Additionally present the values of for the macrocosmic and normal weight babies cross tab.
- Additionally, present Asterix or any other symbol for the significant variables.
- The discussion section is very shallow and try to discuss in detail the possible justification for any similarity or differences in the findings, implication of the findings for community, patients, researchers, and policy makers.
- Try to compare studies conducted in low income set up and specifically Ethiopia and Tigray region.
- What were the strengths of the study?
- Pleas mention in the method that case ascertainment was done by looking the medical record of the mother.
- What efforts are made to control or minimize bias?
