# Supplementary material for: Factors associated with Macrosomia in public hospitals of Mekelle City, Northern Ethiopia: A multi-center study
Source: PLoS One. 2025 Jun 3;20(6):e0325541. doi: 10.1371/journal.pone.0325541 (PMC12132980; doi:10.1371/journal.pone.0325541)
Supplement: Attachment — Submitted filename: A point-by-point response to reviewers.docx [file pone.0325541.s004.docx]

# A point-by-point response to reviewers

Dear Editors and Reviewers, we highly appreciate for your constructive comments and suggestions. Please kindly find our point-by-point response to your precious comments and suggestions.

Reviewer #1:

- Backgrounds demands gaps in previous studies that intended the researcher to carry out the work.
  - Response: We have modified the background/introduction section and added some literatures to enrich it.
- Sampling procedures and techniques: Please have an explanation about number selected for each of the hospital
  - The number of participants from each hospital is determined based on delivery service load in each hospital two-months before the commencement of the study. This statement is already stated in the sampling procedure section.
- Maternal weight, maternal height, weight of the newborn, body mass index (BMI): Please mention the ways the obtain those
  - We have added operational definitions in the method section which will answer how the anthropometric measurements are taken and interpreted.
- For obstetric variables Please ensure that enrolled mothers had under regular Ante Natal Care and booked case in the mentioned hospitals.
  - All participants had antenatal care visit at least once during the first trimester.
- Please make it clear about ways of data collection; is it from hospital record or by interview from mothers/ records from mother
  - The structured questionnaire was used to collect data from the mother through direct interviews, and the checklist was used to extract data from the medical records of both the mother and the newborn. We have added this statement in data collection procedure.
- References:
  9,10,20-23 : Please mention source
  14: Complete reference
  - We have update and corrected the references.

Reviewer #2:

Abstract

- I suggest to mention the cut-off birth weight used to define macrosomia
- In the method section of Abstract, we have added a statement how macrosomia was defined
- I suggest to spell out BMI
  - In Abstract, Now BMI is written as ‘body mass index’

Introduction

- I suggest to insert reference for each sentence or information e.g. line one, line two It is not desirable to cite more than three reference in one place e.g. 1-8. I suggest to explore the introduction more and more. Nothing have been mentioned on diabetes mellites however, it know factor that associated with macrosomia
  - We have made major correction in the introduction/background section and addressed all the concerns and issues related to number of references. Now we have added statements and references related to effect of diabetes on macrosomia
- Statistics
  Were the continuous data checked for normality?
  I think your continuous variables were not normally distributed
  - We have checked for normality. All variables mentioned in the manuscript were normally distributed and had skewness value between -2 to +2. We used this cut-off value based on the book titled “An introduction to statistical concepts” by By Debbie L. Hahs-Vaughn, Richard Lomax, 4th Edition, 2020, <https://doi.org/10.4324/9781315624358>
