# Supplementary material for: Factors associated with Macrosomia in public hospitals of Mekelle City, Northern Ethiopia: A multi-center study
Source: PLoS One. 2025 Jun 3;20(6):e0325541. doi: 10.1371/journal.pone.0325541 (PMC12132980; doi:10.1371/journal.pone.0325541)
Supplement: Attachment — Submitted filename: Comment on macrosomia.docx [file pone.0325541.s003.docx]

**Title:** **Factors Associated with Macrosomia in Public Hospitals of Mekelle City, Northern**

**Ethiopia: A Multi-Center Study**

Dears,

We express our sincere gratitude to the authors for their valuable research on this important public health issue. We also thank the editors for providing us with the opportunity to review this crucial work.

**General Comments:**

While the study provides valuable insights into macrosomia in Mekelle City, a more comprehensive review of existing literature on macrosomia in Ethiopia is crucial. The current discussion primarily compares with studies conducted in other countries (China, Nigeria, etc.). The authors should dedicate a section to discussing the findings of this study in the context of existing Ethiopian research on macrosomia. This would enhance the local relevance and impact of the study. A more thorough literature review on macrosomia in Ethiopia would allow for a better understanding of the unique challenges and risk factors specific to the Ethiopian context.

**Specific Suggestions:**

- Please format your abstract to include the following subheadings are Background, Methods, Results, and Conclusions. Therefore, please remove the Objectives subheading and include the text in one of the above sections.
- The authors should incorporate citations (reference) in some sentences e.g. introduction part paragraph 1 line 2, paragraph 2 lines 2-3.
- Authors should revise paragraphs one and two of the introduction to reduce redundancy. These two sentences convey essentially the same information. To reduce redundancy, the authors could combine the sentences
- In the introduction, the authors should include a dedicated paragraph summarizing key findings from relevant studies conducted in Ethiopia on the prevalence and risk factors of macrosomia
- Inconsistency in how birth weight is categorized in introduction and methods. If there is a valid reason for using two categories (normal and macrosomia) in the methods and analysis, clearly explain this rationale in the introduction or discussion.
- How did the authors screen for pre-existing and gestational diabetes?
- Result section---the sentences “Our study indicated that the mean (SD) age of cases and controls was 33.9(4.7) and 27.5(4.0) years. Maternal age or newborn age represent?
- The discussion section should be expanded to include a comparative analysis of the study's findings with those of other studies conducted in Ethiopia. This could highlight similarities, differences, and potential explanations for any observed discrepancies.
- The authors should discuss the implications of their findings for public health interventions in Ethiopia, considering the specific context and existing healthcare resources.
- Limitation of the study should be discussed, especially the control for preexisting and gestational diabetes which are the very important risk factors for macrosomia
- The authors should also discuss about the generalizability of the study results since the samples were only from mekelle city.
